# Phytochemical Composition and Health Benefits of Figs (Fresh and Dried): A Review of Literature from 2000 to 2022

**DOI:** 10.3390/nu15112623

**Published:** 2023-06-03

**Authors:** Amandeep K. Sandhu, Maria Islam, Indika Edirisinghe, Britt Burton-Freeman

**Affiliations:** Department of Food Science and Nutrition, Center for Nutrition Research, Institute for Food Safety and Health, Illinois Institute of Technology, Chicago, IL 60616, USA; asandhu2@iit.edu (A.K.S.); mislam17@hawk.iit.edu (M.I.); iedirisi@iit.edu (I.E.)

**Keywords:** figs, phytochemicals, anthocyanins, health benefits, processing, extraction, bio-accessibility, diabetes, obesity

## Abstract

With their rich history dating back 6000 years, figs are one of the oldest known plants to mankind and are a classical fruit in the Mediterranean diet. They possess a diverse array of bioactive components, including flavonoids, phenolic acids, carotenoids, and tocopherols, which have been used for centuries in traditional medicine for their health-promoting effects addressing gastrointestinal, respiratory, inflammatory, metabolic, and cardiovascular issues. This review summarizes the updated information on the phenolic composition, antioxidant capacity and other functional properties of fresh and dried figs cultivated in various parts of the world, highlighting variation in phenolic composition based on cultivar, harvesting time, maturity stage, processing, and fig parts. Additionally, the review delves into the bio-accessibility and bio-availability of bioactive components from figs and their potential influence on cardiovascular health, diabetes, obesity, and gut/digestive health. Data suggest that the intake of figs regularly in the diet, alone or with other dried fruits, increases select micronutrient intake and is associated with higher diet quality, respectively. Research in animal and human models of health and disease risk provide preliminary health benefits data on figs and their extracts from fig parts; however, additional well-controlled human studies, particularly using fig fruit, will be required to uncover and verify the potential impact of dietary intake of figs on modern day health issues.

## 1. Introduction

Figs (*Ficus carica*, L.) belong to the Moraceae (mulberry) family, a type of deciduous tree or shrub native to the Middle East and Southwest Asia [1]. The history of figs dates back to the Roman Empire and their significance is also noted in holy books such as the Bible and the Quran [2]. They are believed to be one of the oldest cultivated plants [3] associated with the origin of Mediterranean horticulture. Today, figs are cultivated throughout the world in countries with warm and dry climates.

Figs are commonly known as a fruit, but are actually a type of flower. The fig fruit develops from a closed inflorescence, which encloses hundreds of tiny unisexual flowers. These flowers bloom inside the fig, and the small fruits inside the flowers are what people consume. As a result, figs are considered to be an aggregate fruit, made up of several hundred individual drupelets that form from the ovaries. Fig trees produce two crops per year: the first crop on the previous season’s growth (breba crop) and a second crop on current growth (main crop) [2]. There are four types of fig fruits, i.e., Caprifigs, Smyrna, San Pedro, and Common [4], with over 800 different fig varieties cultivated in almost 50 countries around the world [5]. Turkey was the leading producer of figs (320,000 tons) in 2021, followed by Egypt (298,498 tons) and Morocco (144,153 tons) ranking second and third, respectively. The other fig producing countries that made the top 10 list in 2021 include Algeria, Iran, Spain, Syria, Uzbekistan, USA, and Albania [6].

Figs are a harvest crop for both fresh and dried consumption by humans and a valuable food source for wildlife. Mature edible figs have a thick skin with a sweet pulp consisting of tiny seeds, which are typically unnoticeable but may provide a subtle crunch upon chewing [7]. The skin color in different fig varieties varies from green to black-violet depending upon the pigment compounds present [8]. They are consumed fresh (peeled or unpeeled) and dried, and as part of various foods such as cakes, pies, puddings, bakery products, jams, marmalades, and pastes [3]. More recently, figs are used in sauces complimenting savory meat dishes, mixology creations, and sliced on Mediterranean-inspired pizza, flatbreads and salads. In addition to their culinary versatility, figs have a long history of use in traditional medical practices such as Chinese and Indian (Siddha and Ayurvedic) medicine systems [9]. They have been valued for centuries for their beneficial effects on various health conditions, including gastrointestinal, respiratory, inflammatory, metabolic, and cardiovascular disorders [1]. Figs are an excellent source of bioactive components including vitamins, minerals, organic acids, amino acids, dietary fibers, and an array of phytochemical components, including carotenoids and polyphenolic compounds. However, figs are under appreciated in terms of health benefits compared to other fruits. The phytochemical composition of fruits is a discriminating factor in understanding the health benefits of fruits in the diet.

**Figure 1:**
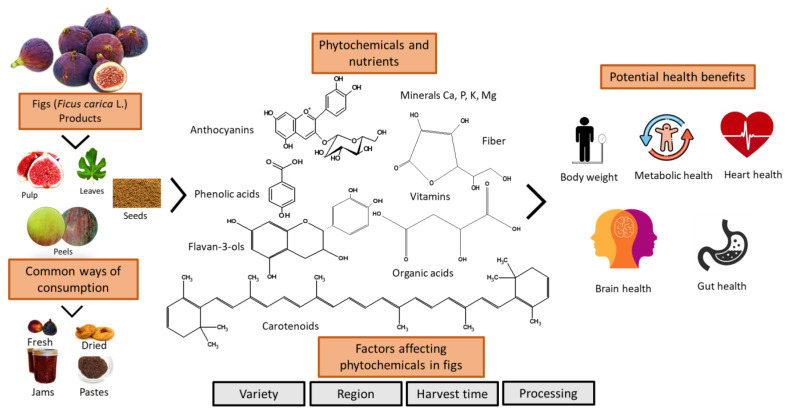
Factors affecting figs’ bioactive compounds and their potential health benefits. Fig image source: Rasool et al. [10]. Health benefits images: stock google photos.

The goal of the present paper is to provide a comprehensive review of the available literature assessing the phytochemical composition and health benefits associated with the consumption of fresh or dried figs, specifically cardio-vascular diseases, diabetes, gut/digestive health, cognitive function, obesity, satiety, and dietary patterns (Figure 1). The purpose of this review is to evaluate the scientific evidence relevant to the chemistry of figs and their potential health-promoting role in the diet, to identify gaps in the current research on figs, and to suggest potential opportunities for future research and development. Research between 2000 and 2022 was identified in Medline with PubMed searches using the keywords provided in Table 1. Searches were also conducted in Web of Science, Google, and by cross-referencing published papers.

## 2. Fig Chemistry

### 2.1. Phytochemical Content of Figs

**Figure 2:**
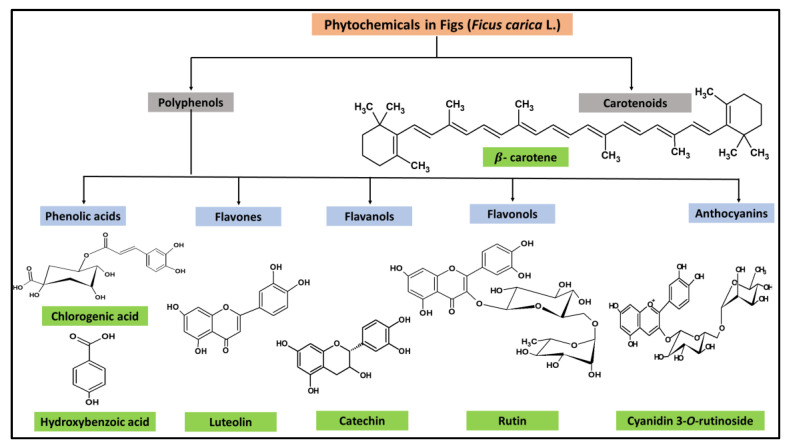
Chemical structures of major phytochemicals in figs.

Polyphenols and carotenoids are the two major categories of phytochemicals found in figs. The major classes of polyphenols in figs include phenolic acids, flavones, flavonones, flavonols, anthocyanins, and proanthocyanidins (Figure 2). The phenolic content of figs is higher than red wine and tea, the two prominent and well-published sources of various phenolic compounds [11]. In addition, the anthocyanin content of some fig cultivars is comparable to blackberries and blueberries [12]. The phytochemical content of figs has been reviewed by other authors [5,13,14]. In this section, we discuss the phytochemical content of figs based on the type of analysis, the extraction of fig polyphenols using various solvents, and the effect of harvesting time and processing on phytochemical composition (Supplementary Table S1).

#### 2.1.1. Types of Analyses and Reported Phytochemical Features

Spectrophotometric analysis: Different fig varieties (dark and light skin colored) as whole or parts (peel, pulp, and leaves) have been compared for their polyphenol content and antioxidant capacity in various geographical locations. The phytochemical content using spectrophotometric assays have measured and reported total phenolic content (TPC), total anthocyanin content (TAC), total flavonoid content (TFC), total proanthocyanidin content (TPAC), total carotenoids, total chlorophylls, total tannins, and ortho-diphenols, as discussed below. Some assays, such as the pH differential method, provide data on specific flavonoid compounds. Totals often predict antioxidant capacity, as many polyphenols and carotenoids possess antioxidant properties. Antioxidant capacities are assessed using various assays such as ORAC (oxygen radical absorbance capacity), DPPH (1,1-diphenyl-2-picryl hydrazyl), FRAP (ferric reducing antioxidant power assay), and ABTS (2,2′-azinobis-(3-ethylbenzothiazoline-6-sulfonic acid). Other assays mentioned include measuring reducing power against hydroxyl, nitrite and superoxide radicals, hydrogen peroxide scavenging activity, cupric ion reducing antioxidant assay (CUPRAC), lipid peroxidation inhibition capacity (LPIC) assay, the thiobarbituric acid reactive substances (TBARs), the oxidative hemolysis inhibition assay and metal chelating activity, phosphomolybdenum, rancimat, and β-carotene blanching assays. The results from various studies suggest varietal differences in polyphenolic content and antioxidant capacity [15,16].

Dark varieties have higher polyphenol content (TPC, TAC, and TFC) and antioxidant capacity compared to lighter varieties [3,17,18,19,20,21,22,23,24,25,26,27,28,29,30,31,32,33,34,35]. A comparison of different parts of figs (leaves, peel, and pulp) revealed that leaves possess high TPC and antioxidant capacity followed by peel and pulp [36,37]. Moreover, Oliveira et al. (2009) reported that only leaves were able to scavenge superoxide radicals compared to peel and pulp [38]. Contrary to this, Mopuri et al. (2018) reported that fig fruits have high antioxidant capacity and phenolic content compared to stembark and leaves [39]. However, parts of the figs were from different regions, possibly explaining the discrepancy in results (e.g., stembark and leaves were collected from India while fruits were purchased from S. Africa). Studies comparing peels and pulps of figs reported that peels have higher concentrations of phenolic compounds and antioxidant capacity compared to pulp, regardless of fig color [18,22,30,40,41,42].

The total anthocyanins by pH differential ranged from 0.41 to 57.47 mg cyanidin-3-*O*-rutinoside/100 g dry weight (DW) in 135 Moroccan fig varieties [35]. In another study on five fig varieties, the total polyphenols content varied from 45.24 to 160.42 GAE mg/100 g DW of the sample, while the anthocyanin content varied less, from 0.0 to 5.32 mg cyanidin-3-*O*-glucoside/100 g DW, and flavonoids from 18.31 to 36.95 mg (+) catechin/100 g DW of the sample. The antioxidant capacity was significantly different between light and dark cultivars [22], which corresponded to the total polyphenols and total anthocyanins [30]. Another study reported that black cultivars had a 2-fold greater total antioxidant capacity, 15-fold greater TAC, and 2.5-fold greater TPC than green and yellow fig cultivars [25]. In comparison to other dried fruits consumed in Algeria, such as apricots, prunes, and raisins, figs had the highest concentration of flavonoids (105.6 mg QE/100 g; QE-quercetin equivalents) and anthocyanins (5.9 mg/100 g) while apricots along with figs had the highest concentrations of carotenoids (10.7 and 10.8 mg βCE/100 g, respectively; βCE-beta carotene equivalents) [43].

High performance liquid chromatography (HPLC) analysis: Phytochemicals in figs (leaves, fruits, peel, and pulp) have been identified and quantified more selectively and comprehensively using HPLC coupled with mass spectrometry (MS) and other detectors such as diode array detector (DAD), Ultraviolet/Visible (UV/Vis), photodiode array (PDA), etc. The polyphenolic compounds identified in most studies on figs are categorized as flavan-3-ols, phenolic acids, flavonols, flavones, and anthocyanins [12,44]. Several studies have reported that skin/peel had the highest concentration of phenolic compounds compared to pulp [12,26,27,45], and that the color of the figs influences their composition and could affect the concentration of phenolic compounds [26,27,29]. In addition, processing could influence the concentrations of phenolic compounds [46]. The major phenolic compounds identified and quantified in figs/parts include quercetin-3-*O*-rutinoside (rutin), (−)-Epicatechin, (+)-catechin, cyanidin-3-*O*-rutinoside, bergapten, myricetin, and kaempferol, along with various phenolic acids [15,24,38,42,46]. However, gallic (1.5–6.4 mg/100 g FW) and ellagic acids (0.2–33.8 mg/100 g FW) as major phenolic acids were reported in four Georgia (USA) grown fig cultivars [47]. Phenolic acids, such as 2,4-dihydroxybenzoic, 2,3-dihydroxybenzoic and sinapic acid, were identified for the first time in fig cultivars from Greece [48]. In addition to previous reports on phenolic compounds, Vallejo et al. (2012) reported the c-glycosides of flavones (luteolin 6C-hexose-8C-pentose) for the first time in a study conducted on 18 fig varieties from Spain [12].

Several studies have focused on the pigment chemistry of figs. Anthocyanins, flavonols, and carotenoids are the main pigment compounds in figs as measured by HPLC methods. A recent study conducted by Hssaini and co-workers (2021) investigated polyphenols in 25 fig varieties grown in Morocco and quantified 12 phenolic compounds in peel and 8 in pulp [42]. Anthocyanins, mainly cyanidin-3,5-diglucoside, and cyanidin-3-*O*-rutinoside, were the predominant compounds in peels, with mean concentrations of 75.90 ± 18.76 and 77.97 ± 18.95 µg/g dry weight (DW), respectively. In addition, pelargonidin-3-*O*-rutinoside was detected in the peels. (−)-Epicatechin (a flavanol) and cyanidin-3-*O*-rutinoside were the major compounds in the pulp extracts, where the mean values were 5.23 ± 4.03 and 9.01 ± 5.67 µg/g DW, respectively. Similarly, a study conducted on one fig variety from Portugal quantified 15 phenolic compounds in peel with rutin (flavonol: quercetin-3-*O*-rutinoside and sophorin) as the major constituent and 12 in the pulp with caffeic acid derivatives as major constituents [40]. A study conducted by Ammar et al. (2015) characterized 116 phenolic compounds in the leaves, fruit, skins, and pulps of two fig cultivars (green and black) from Tunisia, and reported that the leaves and the skin of black cultivars had a rich qualitative polyphenolic profile, and that rutin was the main component in fruits, skins, and leaves, while prenylhydroxygenistein was the major component in pulp. A total of 9 anthocyanins were characterized, with cyanidin 3-*O*-rutinoside and cyanidin 3,5-diglucoside as the major ones, two of which were detected in green cultivars [49]. Rutin has been identified as a major compound in various fig varieties from different geographical regions [49,50,51,52]. Research also suggests that the amount of rutin in figs is comparable to apples, with the highest concentration of rutin up to 28.7 mg per 100 g of fresh weight as reported in three fig cultivars [29]. Comparison of 19 fig varieties from three different geographical regions (Italy, Turkey, and Greece) in fresh and dried forms showed significant quantitative and qualitative differences in phenolic compounds [45]. Dueñas et al. (2008) studied the anthocyanin composition in five fig varieties (green and dark purple) from Spain and identified 15 anthocyanin pigments with cyanidin as the major aglycone, and some pelargonidin derivatives were also detected [53]. They also found rutinose and glucose as the major sugars attached to aglycones, and observed acylation with malonic acid. In addition, they also reported anthocyanin-derived pigments such as 5-carboxypyranocyanidin-3-rutinoside, a cyanidin 3-rutinose dimer, and five condensed pigments containing C–C linked anthocyanins (cyanidin and pelargonidin) and flavanol (catechin and epicatechin) residues. The peel had higher concentrations of anthocyanins than the pulp, with cyanidin-3-*O*-rutinoside and malonyl derivatives present in higher amounts in the peel. Total anthocyanin content in the peel ranged between 32 and 97 μg/g and between 1.5 and 15 μg/g in the pulp [53]. Other studies have also found cyanidin-3-*O*-rutinoside as the major anthocyanin in different fig varieties [27,30,48,49,52,54,55]. However, a study conducted on six fig cultivars in China reported cyanidin-3-*O*-glucoside as the major anthocyanin, followed by cyanidin-3-*O*-rutinoside and cyanidin-3,5-diglucoside [56]. A study from Greece also found delphinidin-3-*O*-glucoside, petunidin-3-*O*-glucoside in the pulp, and malvidin-*O*-glucoside in both the pulp and peel for the first time in fig varieties [48].

Carotenoids found in figs include lutein, zeaxanthin, β-cryptoxanthin, and β-carotene. Yemis et al. (2012) identified these pigments in yellow fig varieties and found that the surface color of fig fruits changes with the ripening stage [54]. In a study conducted to evaluate the carotenoid composition in selected foods of the Mediterranean diet, figs were reported to contain all the major carotenoids including lutein, β-carotene, α-carotene, cryptoxanthin, and lycopene [57]. Tocopherols were also detected in fig cultivars [40,47]. Palmeira et al. detected all four forms (α, β, δ and γ) of tocopherols in one Portuguese fig variety with α tocopherol abundant in peel and γ tocopherol abundant in pulp [40].

Overall, both green and dark varieties contain anthocyanin pigment compounds, with cyanidins reported most consistently with rutinoside and glucoside sugar attachments. Additional anthocyanin structures, particularly those imparting reds and dark blue and purple hues, have also been reported. Rutin and carotenoids are other important pigment compounds in figs. An array of phenolic acids as well as other flavonoid compounds, such as catechin and epicatechin have been reported, which collectively, and uniquely, characterize the polar fraction of figs.

Extraction of polyphenols from figs: Different methods and solvents have been compared for extracting polyphenols from figs. These methods include using various solvents (water, acetone, ethanol, methanol with or without acids), altering time–temperature combinations, and using ultrasound-assisted extraction and high-pressure processing [58,59,60,61,62,63,64,65,66]. Studies have optimized the extraction conditions for the maximum recovery of phenolic compounds from figs, with some reporting that double extraction with 60% acetone without acidification at 40 °C for 120 min with a 1/75 solid to solvent ratio was optimal [64]. The same research group used response surface methodology to further optimize the conditions [67]. Similarly, Mezziant et al. optimized the extraction of anthocyanins from fig peels and observed that double extraction with 90% methanol acidified to a ratio of 10/90, with 5% citric acid using a solid-to-solvent ratio of 1/100 extraction time, for 180 min yields the maximum concentration of anthocyanins from dried fig peels [62]. Additionally, some studies have compared the use of different solvents (acetone, ethanol, methanol, and water) for the extraction of phenolic compounds from figs [51,59,60,65]. A recent study conducted by Tewari et al. comparing the extraction of phenolic compounds from wild Himalayan fig varieties using various solvents (methanol, boiling water, and Soxhlet with methanol), showed significant variability among the extraction solvents. Water extracts of figs had the highest antioxidant capacity, while methanol extracts had a better ability to inhibit enzymes and more compounds were identified in the methanol extract [60]. Other studies have used ultrasound-assisted extraction (UAE) alone or in comparison to other extraction methods, such as solid-liquid extraction (SLE), heat, and microwave extraction in different fig varieties and parts, and found that phenolic compounds were highest in UAE [61,63,66]. The purification of phenolic compounds from figs has also been conducted using an aqueous two-phase system, resulting in higher antioxidant capacity and a higher concentration of specific compounds (chlorogenic acid, rutin, catechin, and epicatechin) compared to crude extracts [58].

#### 2.1.2. Factors Influencing Phytochemical Composition of Figs

Effect of harvesting time on polyphenol content of figs: The effect of crop harvesting time on polyphenol content has also been reported for different fig varieties. For example, some studies have reported fruits from the first crop (breba) as being richer in phenolic compounds than the second crop (main crop) [12,45]. However, a study conducted by Hoxha et al. on two Albanian fig varieties reported that the main crop of both varieties had higher total phenolic content than the breba [31]. Another study reported neutral effects of crop harvesting time on polyphenols [28]. A study conducted by Gündeşli et al. (2021) compared phenolic compounds in one Turkish fig variety at four different harvesting periods and observed phenolic content to be highest at the first harvest and lowest at the fourth harvest [24]. A similar decrease in total phenolic content and antioxidant capacity with fruit development was observed in a recent study conducted on two Albanian fig varieties [68]. However, Marrelli et al. reported an increase in the amounts of polyphenols with the ripeness of figs [69]. Another study investigated physio-chemical changes in the fig and in the dry fruit during four developmental stages including, three weeks after full bloom; three days after caprification; ripening; and the onset of fruit drying on the tree. The total phenolic compounds decreased until the ripening stage and then increased until senescence, while some phenolic compounds ((+) catechin, chlorogenic acid, (−) epicatechin, and quercetin-3-*O*-glucoside) were highest when fruits dried on the trees [70]. Zhang et al. reported no change in the accumulation pattern of anthocyanins (cyanidin-3-*O*-glucoside and cyandin-3-*O*-rutinoside) at different ripening stages [56]. Likewise, during ripening, no changes were observed in the phenolic compounds, total carotenoid content, and antioxidant capacity of fig pulp from three different varieties; however, anthocyanins increased in the pulp of all cultivars with ripening [48]. A comprehensive study on changes in fig color at different maturity stages using metabolomic and transcriptomic analyses showed significant variation in the accumulation of various flavonoids, including anthocyanins, at young and mature stages due to the upregulation/downregulation of genes involved in the flavonoid biosynthesis pathway [71]. Overall, harvest time and ripening stage influence phytochemical content. With the goal of harvesting figs with the densest phytochemical profile and content, systematic research to identify target periods in different growing regions for different varieties will be required.

Effect of processing on polyphenol content of figs: Fresh figs are extremely perishable and, as a result, are typically processed into various forms such as dried, jams, jellies, nectar, etc. The most common preservation method of fresh figs is drying them. Various studies have investigated the effects of different drying modes (sun, oven, microwave, greenhouse, etc.) on the phytochemical composition of figs. Two studies reported no differences in drying method (sun or oven) on fig polyphenols [20,72], while two other studies reported that sun drying impacts fig polyphenol content, including decreased phenolic acid content by ~29% and flavonoid content by about 86% [73], and reduced total phenolics, total anthocyanins, and antioxidant activity [52]. A comparison of sun, hot-air oven, and microwave drying methods in one fig variety revealed microwave drying to be the best drying method for preserving the polyphenol content [74]. Similarly, the quality of two fig varieties from Tunisia was compared after open air and greenhouse drying. The greenhouse dried figs had twice the amount of total phenolic content compared to open air-dried figs. However, levels of trace elements (Mn, Fe, Cu, and Zn) decreased after the greenhouse drying of the figs [75]. 

Figs may also be frozen or prepared into jams and nectars. Keeping figs in the freezer too long may cause some bioactives’ degradation. The processing of figs into jam could help to preserve some of the phenolic compounds and carotenoids as indicated by research on fresh, frozen, and processed figs [76]; however, other researchers have reported opposite results [77]. Overall, processing technologies are necessary to extend the culinary and nutritional contributions of figs globally. Innovations in processing technologies relative to variety and regions have grown to preserve and optimize nutrient and polyphenol content, including the bioavailability of their compounds, which is an area for future research.

The timing of harvest and processing of figs influences their phytochemical content. Other important factors include variety, growing regions, and agronomic practices. Recent advances in analytical chemistry and nutritional sciences have revealed bioactive features of fruits that describe their dietary value. The fig may be a forgotten fruit in some cultural cuisines; however, evidence-based, consumer-driven health trends may find an old fruit resurrected, delivering nutritional and phytochemical content promoting human health.

### 2.2. Nutrients in Figs

Figs are a rich source of various micro and macronutrients including carbohydrates, vitamins, organic acids, dietary fiber, and minerals [78]. Proximate composition analysis shows that figs are high in protein (6.31 g/100 g (dry weight basis, DW)) and fiber (17.81 g/100 g, DW), with fat content varying from 1.02 to 2.71 g/100 g DW in edible wild fig fruits [36,79]. Different fatty acids have been characterized in various fig varieties with linolenic reported as the most abundant followed by linoleic acid, palmitic, and oleic [17,40,69,78]. Figs also contain high amounts of carbohydrates (26.02 ± 0.63 g/100 g fresh weight) [41,80] and amino acids, such as leucine, lysine, valine, and arginine [80]. Additionally, figs contain organic acids, sugar, and minerals which are discussed in detail below.

Organic acids and sugars: Organic acids and sugars in different varieties of figs have been analyzed in various studies using HPLC [25,29,72,81,82,83]. Organic acids and sugars are high in dried figs compared to fresh figs [72]. Palmiera et al. reported four free sugars (glucose, fructose, trehalose, and sucrose) and five organic acids (oxalic, quinic, malic, citric, and succinic acids) in the peel and pulp of one Portuguese fig variety [40]. In a study conducted on 9 fig varieties from Spain, the concentrations of sugars were highest in the pulp, followed by the skin/peel, with no differences observed in organic acids between varieties and ripening stages [82]. A comparison of 27 Tunisian fig varieties showed significant differences in glucose and fructose content [32]. The major organic acids studied in fig fruits or their parts include malic, citric, oxalic, quinic, ascorbic, shikimic, and fumaric acids [16,38,40,47,70]. It has also been reported that the accumulation pattern of organic acids changes at various ripening stages [56]. The taste and flavor profile of fruits is determined by the ratio of organic acids to sugars. Organic acids are essential for preserving the nutritional value and enhancing the sensory qualities of foods. They also provide various health benefits, including reducing inflammation, regulating the immune system, promoting calcium absorption, and preventing blood clots [84].

Minerals: Figs have the highest mineral content compared to other common fruits [11]. Figs are an important source of potassium, calcium, sodium, magnesium, phosphorous [41,79,80,85,86], and trace elements such as iron, manganese, zinc, copper, nickel, and strontium [70,79,86].

### 2.3. Bio-Accessibility and Bioavailability of Phytochemicals from Figs

Bio-accessibility refers to the proportion of a nutrient in a food that becomes available for direct absorption or biotransformation by gut microbiota during the process of digestion. Bioavailability, on the other hand, describes the proportion of an ingested nutrient that is absorbed and reaches systemic circulation or specific tissues and organs in the body, in its intact or metabolized form. These absorbed nutrients or phytochemicals can then exert a biological action or be stored for future use [87]. The phenolic compounds from figs are not readily bio-accessible, as reported by various studies using in vitro gastrointestinal digestion models [52,88,89]. For example, a study conducted by Kehal and colleagues, using three fig cultivars in fresh and dried forms, showed that phenolic compounds and antioxidant capacity decreased during different digestion phases (oral phase > gastric phase > intestinal phase). The study also found that sun-drying and cultivar had no impact on the in vitro digestion of phenolic compounds and antioxidant activity from figs [88]. Alternatively, Kamiloglu and colleagues (2013) reported that the sun-drying of figs results in an increased bio-accessibility of total proanthocyanidin and chlorogenic acid content, as well as total antioxidant activity, compared to fresh figs; however, the bio-accessibility of anthocyanins (cyanidin-3-*O* glucoside and cyanidin-3-*O* rutinoside) was very low for fresh figs and anthocyanins were not detected in the dialyzed fraction of sun-dried figs [89]. A similar study by Kamiloglu et al. (2015) reported a reduced bio-accessibility of phenolic compounds from figs with a higher value of phenolic compounds in the dialyzed fraction obtained from the skin compared to the pulp of all the studied varieties [52]. The variations in bio-accessibility of different components can be attributed to several factors. One possible reason is the susceptibility of these phenolic compounds to enzymes and changes in pH during the process of digestion. For example, anthocyanins could be transformed to colorless chalcones at pH 7, which might not be detectable by the methods employed [90]. The increased bio-accessibility of some components from dried figs could be explained by the higher concentrations of phenolic components per unit weight of dried figs compared to fresh figs which have more water. Overall, the bio-accessibility and bioavailability of nutrients can vary depending upon various factors such as cultivar, processing, interaction with other dietary components and inter-individual variations influenced by host genes, and those from the composition of the gut microbiome [91]. There are no studies on the absorption, metabolism, and bioavailability of fig polyphenols in humans. Only one human study [92] conducted in the United States reported plasma antioxidant activity in normal free-living participants (*n* = 10) after the consumption of 40 g of figs with or without a carbonated beverage. The plasma antioxidant capacity was measured for six hours using the Trolox equivalent antioxidant capacity (TEAC) assay. The authors reported increased plasma antioxidant capacity for 4 h after consumption of figs and reduced oxidative stress generated by consuming high fructose corn syrup in a carbonated soft drink.

Figs are a diverse fruit that can vary in phytochemical and nutrient composition based on factors such as location, variety, harvesting time, and ripeness. Different analytical methods reveal different features of the content of fruits, including figs. Consistently, figs are reported to contain phenolic compounds, such as anthocyanins, (−)-epicatechin, rutin, and chlorogenic acid, among others, which, over the last two decades, have accumulated evidence suggesting they impart biological activity when consumed by humans. However, there is currently a lack of research on the bioavailability and absorption of these compounds when figs are consumed as part of a regular diet. Further studies on the pharmacokinetics of phenolic compounds from figs in conjunction with their potential health benefits, would be valuable for dietary guidance.

### 2.4. Figs Health Benefits

In the previous section, we provided an in-depth discussion on the phytochemicals and nutrients present in figs, as well as their bio-accessibility and bioavailability. It is worth noting that the literature extensively covers the phytochemistry of figs grown in regions outside of the USA. There also is a lack of comprehensive information regarding the consumption of figs as a source of dietary phytochemicals for human beings. Taken together, there is a need for future research to investigate figs grown in the USA, including the bioavailability of key phytochemicals in figs following their ingestion.

**Figure 3:**
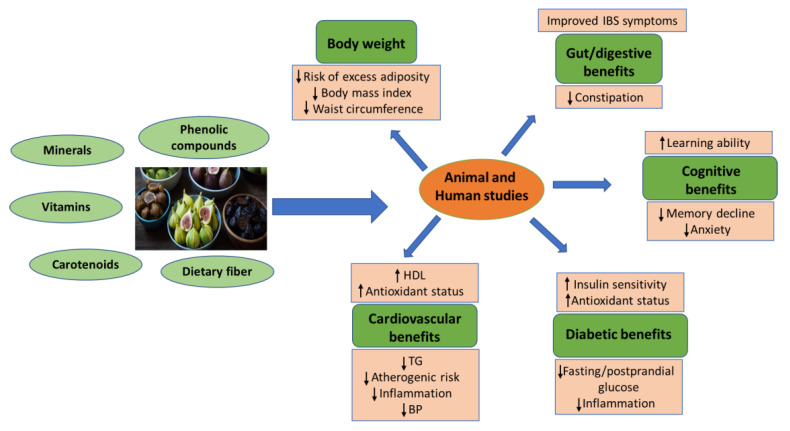
Summary of health benefits of figs from animal and human studies. Abbreviations: BP, blood pressure; HDL, high-density lipoprotein; IBS, irritable bowel syndrome; TG, triglycerides. Arrows: ↑ (increase), ↓ (decrease). Fig image source: California Fig Advisory Board (https://californiafigs.com/, accessed on 25 May 2023).

Epidemiological and clinical studies provide evidence suggesting that phytochemicals/bioactives from fruits and vegetables exert beneficial effects on human health post-consumption. Building upon this knowledge, this section on figs’ health benefits will review the available research conducted on both animals and humans. We will explore the effects of different fig components, including the flesh/pulp, juice, peel, extract, dried, and fresh forms, on various health risk conditions such as cardiovascular diseases, diabetes, obesity, cognitive function, and gut/digestive health, as well as the impact of figs on satiety and dietary patterns (Table 2, Table 3, Table 4, Table 5 and Table 6 and Figure 3).

#### 2.4.1. Cardiovascular Risk Benefits

Cardiovascular diseases (CVD) are a group of metabolic disorders of the heart and the blood vessels. The most important behavioral risk factors of cardiovascular diseases are modifiable, including unhealthy diet and physical inactivity. The effects of behavioral risk factors may show up in individuals as increased blood pressure, blood glucose, blood lipids, and overweight/obesity. Limited data are available assessing the relationship between figs and CVD risk and the majority of them are in animal research (Table 2). In humans, fig intake on CVD risk factors was assessed in individuals who were overweight and had one CVD risk factor or individuals with elevated cholesterol [93] or who had rheumatoid arthritis [94] (Table 2). Fig intake may be consumed as part of a dried fruit mix, as was the case in one study incorporating ¾ cup per day dried fruit vs. a carbohydrate-rich snack for 4 weeks [95]. An earlier study by Peterson et al. (2011) fed individuals 120 g/d California Mission figs for 5 weeks [93]. Research from both groups indicated no changes in body weight throughout the study and changes in lipids (HDL; high density lipoproteins and TG; triglycerides) were mostly unaffected. However, a sequence effect suggested that cholesterol levels increased if fig intake was initiated first in the crossover study [93]. Low density lipoprotein (LDL) and fasting glucose were increased in the dried fruit mix study [95]. No effect on lipids or glucose was found in patients with rheumatoid arthritis medication regimens containing methotrexate [94].

In the animal literature, fruit extract [96,97,98], leaf extract [98,99], and seed oil [100] were tested. The fruit extract decreased blood pressure in normotensive and glucose-induced hypertensive rodents after 3 weeks. Furthermore, researchers found blood pressure reduced during the first 1–3 h after dosing (1000 mg/kg), returning to baseline by 6 h [96]. Anti-inflammatory and anti-oxidative activity were demonstrated in an intestinal ischemia-perfusion injury model and in a high-fat-fed obesity model with seed oil and leaf extracts [98,99,100]. The latter research also reported increased HDL, decreased TG, and overall reduced atherogenic risk after 6 weeks of supplementation with fig leaf extract [99]. Overall, animal research reveals the important CVD-promoting effects of figs; however, to date, these effects have not been observed in human research, and there is extremely limited data from which to draw conclusions.

**Table 2:** Cardiovascular risk benefits.

Study Details		Intervention				Results			
First Author Year	Study Type Design	Population Model	Sample Size	Duration	Fig (Tx)	Control (Tx)	Blood Pressure	Lipids	Other
Human Research									
Sullivan, VK, 2020 [95]	RCT crossover	Overweight + 1 risk factor	55	4 weeks	Fig as part of dried fruit mix 3/4 c snack	high carb snack	↔ BP	↔ lipids/ lipoproteins btn Tx ↑ LDL within dried fruit arm	↑ fasting glucose ↔ insulin ↔ vascular stiffness ↔ CRP
Bahadori, S, 2016 [94]	RCT Parallel	Arthritis ~51 y	56 29:27	16 weeks	Fig + OO	DMARDs		↔ TC ↔ TG ↔ LDL ↔ HDL	↔ Glucose
Peterson, J, 2011 [93]	RCT Cross over	30–75 y TC 100–189 mg/dL	83 41:42/ seq	5 weeks	Fig CA Mission 120 g/day	Usual diet w/o Fig		↑ TC (seq effect) ↔ LDL ↔ HDL ↔ TG	↔ BW ↑ fiber ↑ sugar
Animal Research									
Orak, C, 2021 [100]	Parallel in vivo animal	Albino ischemia-reperfusion injury (IRI) rat model	50	10 days	Fig seed oil 3 mL/kg/d 6 mL/kg/d	Neg control Sham control			Anti-Inflammation ↓ TNFα ↓ IL-1β Anti-Ox ↓ MDA ↓ MPO ↓ histopathology of intestinal tissue
Elghareeb, MM, 2021 [97]	Parallel in vivo animal	chemo-induced Ox stress rat model	40	30 Days	Fruit extract	Vehicle			blunted chemo-induced toxicity on CVD markers
Sukowati, YK, 2019 [98]	Parallel in vivo animal	high fat diet (HFD)-induced obese rat model	32 8/group	10 weeks	Fruit Leaf extract 400 mg/kg	Control diet		↓ lipids (panel)	↓ TNFα ↓ MDA
Alamgeer, IS, 2017 [96]	Parallel in vivo animal	normo- and glucose-induced hyper- tensive rat model	3/group	3 weeks	Fig fruit extract 250, 500, 1000 mg/kg	Vehicle	↓ BP (1000 mg/kg) normo- and hyper- tensive		Phenolic analysis: presence of quercetin, gallic acid, caffeic acid, vanillic acid, syringic acid, coumaric acid, chromotropic acid.
Joerin, L, 2014 [99]	Parallel in vivo animal	high fat diet (HFD)-induced obese rat model	10/group	6 weeks	Leaf extract (FLE) 50 mg/kg FLE 100 mg/kg FLE 30 mg/kg Pioglitazone	Chow and HFD		↑ HDL ↓ TG ↓ IL-6 FLE > Pioglitazone	↓ AI ↓ CRI ↔ adiponectin ↔ leptin ↔ insulin ↔ glucose

Arrows: ↑ (increase) ↓ (decrease) ↔ (no effect). AI: atherogenic index, BP: blood pressure, BW: body weight CRP: C reactive protein, CVD: cardiovascular disease, CA: California, CRI: coronary risk index, DMARDS: disease modifying anti rheumatic drugs, FLE: fig leaf extract, HFD: high fat diet, HDL: high density lipoprotein, IR: ischemia-re perfusion injury, IL-6: interlukin-6, IL-1β: interleukin-1- beta, LDL: low density lipoprotein, MDA: malondialdehyde, MPO: myeloperoxidase, Neg ctrl: negative control, OO: olive oil, Ox: oxidative, RCT: randomized control trial, Sham: sham-operated, TC: total cholesterol, TG: triglyceride, TNFα: tumor necrosis factor alpha, w/o: without.

#### 2.4.2. Diabetes Benefits

Diabetes mellitus (DM) is one of the most common chronic diseases in the world, with a rapidly increasing incidence. Fig plants and their active compounds have been used to treat diabetes and related chronic disorders since ancient times. However, there are only five human clinical research studies and eleven in vivo animal research studies found in the peer-reviewed literature over the last two decades evaluating their anti-diabetes effects (Table 3). Animal studies are mostly focused on the mechanism of actions, and several of those focus on extracts of the leaves vs. investigating the effect of the fruit.

An ethnobotanical survey of medicinal plants conducted by Barkaoui et al. (2017) indicated that the fruits and leaves of figs are used by practitioners to treat diabetes complications in Morocco [101]. Bio-efficacy testing in humans shows the decoction of leaves effectively controlled postprandial glycemia in individuals with T1DM (type 1 diabetes mellitus) [102]. Furthermore, a study by Mazhin et al. (2016) showed that the addition of a fig leaf decoction, compared to oral hypoglycemic drugs, significantly decreased 2 h postprandial glycemia in patients with T2DM (type 2 diabetes mellitus) [103]. In another study, the effect of fig was compared with the oral drug metformin in people with T2DM [104] and this showed that metformin decreased blood sugar levels by 27.6% and figs decreased blood sugar levels by 13.5% after 2 months of treatment. The study concluded that figs decrease blood sugar/glucose levels significantly and, when compared to metformin, this change is about half that of metformin [104].

Studies indicate that abscisic acid (ABA) can improve glucose homeostasis. Figs are an intermediate source of ABA. Avocado have ~2.0 mg/kg ABA, whereas many fruits contain only ~0.3 mg/kg ABA while figs contain ~0.72 mg/kg ABA [105]. A study conducted by Atkinso et al. (2019) showed that two fig fruit extracts (FFEs), each administered at two different ABA doses to healthy human adults, significantly reduced postprandial glycemia at the higher dosages tested [106]. Furthermore, they showed that peak insulin concentrations were significantly reduced by FFE-containing test drinks compared to reference drinks [106]. Zangara and colleagues reported similar findings with a fig fruit extract standardized on ABA in healthy subjects [107]. The glucose- and insulin-lowering action demonstrated in healthy individuals may be clinically important for people with hyperinsulinemia and insulin resistance to lower their risk of T2DM. To test this idea, Leber et al. (2020) studied fig fruit extract of ABA in diet-induced obesity (DIO) and db/db diabetes mouse models, and found improved glucose tolerance, insulin sensitivity, and fasting blood glucose [108]. Furthermore, they showed a decrease in systemic inflammation in response to fig fruit extracts of ABA.

An in vivo rat study, conducted by Irudayaraj et al. in 2016, demonstrated that ficusin, isolated from the leaves of *F. carica*, significantly decreased blood glucose concentrations and improved the lipid profile, plasma insulin, nephrotic markers, liver glycogen, liver enzymes, and protected β-cells. By exploring the mechanism of action, the authors demonstrated that ficusin effectively upregulated PPARγ, and activated glucose transport through translocation and GLUT4 activation in adipose tissue [109]. In a follow-up publication, Irudayaraj et al. (2017) showed that ethyl acetate extract of fig leaves significantly promoted hypoglycemic and hypolipidemic activities in a rat model of T2DM. They demonstrated the altered activities of key carbohydrate-metabolizing enzymes such as glucose-6-phosphatase, fructose-1,6-bisphosphatase, and hexokinase in the liver tissue of DM rats that were improved with fig leaves extract supplementation and comparable to normal levels [110]. Kawther et al. (2009) investigated the hypoglycemic effect of the orally administered aqueous extract of fig leaves in alloxan-induced diabetes in rabbits. Data obtained from the first experiment showed that 0.3 gm/kg body weight of aqueous extract of fig leaf extract given alone or in combination with insulin improved blood glucose levels in diabetic rabbits compared to untreated diabetic rabbits. The results from their second experiment showed there were no significant differences between 8 U/kg insulin and 0.3 g/kg fig leaf aqueous extract group compared to 10 U/kg insulin; furthermore, they showed that the reduction in insulin dose was almost 20% produced by fig leaves aqueous extract [111]. Perez et al. (2000) demonstrated the antidiabetic activity of aqueous extracts from the leaves of *F. carica* in streptozotocin (STZ-induced diabetic rats) [112]. Arafa (2020) showed that fresh seeds and fruit extract from figs reduced serum glucose in high-fat-fed and STZ-induced diabetes rat models [113]. El-Shobaki et al. (2010) reported that raw fig fruits and leaves have antidiabetic activity in alloxan-induced diabetic rats by increasing antioxidant levels [114]. Kurniawan and colleagues recently reported similar findings with leaf extract in the alloxan-induced DM model [115]. In addition, methanol and ethanol extracts from the fig leaves have been shown to reduce blood glucose concentrations significantly in alloxan-induced diabetic rats [116]. Ajman M et al. (2016) also demonstrated that the leaf extract of figs was effective in reducing the blood glucose level in Sprague Dawley rats [36].

**Table 3:** Diabetes benefits.

	Study Details				Intervention		Results		
First Author Year	Study Type Design	Subject Detail	Sample Size	Duration	Fig (Tx)	Control (Tx)	Insulin	Glucose	Other
Human Research									
Atkinson, FS, 2019 [106]	RCT Parallel	Healthy	10	Acute	Fig fruit extract (FFE) in glucose drink standardized to ABA 100 mg 200 mg 600 mg 1200 mg	Glucose Drink	↓ Insulin dose-dependent	↓ Glucose at highest doses	
Shah, M, 2019 [104]	RCT Parallel	T2DM	50 25/group	2 months	Fig 10 g 3.3 g thrice/d	Metformin		↓ Glucose	
Zangara, A, 2018 [107]	Cross Over dose response	Healthy	10	Acute	Extract Glucose solution + 40 or 80 µg ABA in 250 mL water 100 mg ABAlife = 40 µg ABA	Glucose solution (50 g)	↓ Insulin index	↓ Glycemic index	
Mazhin, SA, 2016 [103]	RCT	T2DM	28	21 Days	Fig 13 g of leaf powder	green tea		↓ Glucose OGTT response	
Serraclara, A, 1998 [102]	RCT	T1DM subjects	10 5/group	1 month and post prandial (PP) and fasting	Leaf extract	Non- sweet tea	↓ Insulin exog need PP	↓ Glucose PP ↔ Fasting	
Animal Research									
Leber, A, 2020 [108]	Parallel in vivo animal	DIO and db/db mouse model	10	12 weeks	FIG 0.125 µg ABA/kg BW	Vehicle (water)	↑ Insulin Sensitivity	↓ Glucose fasting ↑ Gluc Tol	↓ TNFα ↓ MCP ↓ IL-6 ↑ metabolic capacity of muscle cells
Kawther, M, 2009 [111]	Parallel in vivo animal	Alloxan—induced DM + High fat diet (HFD) rabbit model	48	6 weeks	Leaf extract 0.3 gm/kg extract +/− insulin	Insulin	↓ Insulin exogenous need	↓ Glucose	
Kurniawan, MF, 2021 [115]	Parallel in vivo Animal	Alloxan-induced DM rat model	8/groups	14 days	Leaf extract 40, 60, 80 mg tablet formula	Placebo Metformin as + control		↓ Glucose	
Arafa, et al., 2020 [113]	Parallel in vivo animal	diabetes via STZ + High fat diet (HFD) rat model	6/group	8 weeks HFD 3 week before start treat for 5 weeks	Seeds and Fruit extract 250 mg/kg/d 500 mg/kg/d	Control no extract		↓ Glucose	↓ BW ↓ TC ↓ TG ↓ LDL ↓ VLDL ↑ HDL Anti-Ox ↑ SOD ↓ MDA
Irudayaraj, SS, 2017 [110]	Parallel in vivo Animal	diabetes via STZ + High fat diet (HFD) rat model	6/group	28 days + OGTT + ITT on 15th and 25th days	Leaf extract 250 mg/kg 500 mg/kg	Vehicle Normal rat and DB rats	↓ Insulin ITT response	↓ Glucose fasting ↓ OGTT response	↓ TC ↓ TG ↓ BW ↓ Glycogen Liver carbohydrate enzymes normalized in DM rats
Ajmal, 2016 [36]	Parallel in vivo animal	normal/wild type rat model	80 10/group	56 days	Fig fruit peel, pulp and leaves	Control diet	↑ Insulin	↓ Glucose Leaf extract	Fig peel, pulp, leaf ↑ fiber ↑ protein ↑ minerals ↑ phenolics ↑ flavonoids ↑ Antioxidant properties
Irudayaraj, SS, 2016 [109]	Parallel In vivo Animal	diabetes via STZ + High fat diet (HFD) rat model	6/group	28 days	Leaf extract of Ficusin 20 mg/kg 40 mg/kg	Vehicle Normal rat and DB rats	↓ Insulin	↓ Glucose fasting ↓ OGTT response	↓ TC ↓ TG ↓ FFA ↓ BW ↓ SOD ↓ Cat ↑ GLUT 4 ↑ PPARγ adipose tissue
Stalin, C, 2012 [116]	Parallel in vivo Animal	Alloxan—induced DM rat model	5/groups	21	Fig fruit extract 100 and 200 mg/kg p.o.	Metformin 500 mg/kg p.o		↓ Glucose fasting	↓ TG fasting
El-Shobaki, FA, 2010 [114]	Parallel in vivo Animal	Alloxan-induced DM rat model	48 6/group	4 weeks	Fig Fruit and Leaf extract 5, 10 and 20% fruit 4, 6, 8% leaf extract	Control diet		↓ Glucose fasting in DM	↓ lipids Liver and Kidney Fxn improved
Perez, C, 2000 [112]	Parallel in vivo Animal	non-DM and DM STZ-induced DM rat model	52 13/group	3 weeks	Leaf extract 2.5 g/10 mL	Water	↓ Insulin non-DM ↔ DM	↓ Glucose fasting in DM ↔ Non-DM	

Arrows: ↓ (decrease), ↑ (increase), ↔ (no effect). Alloxan induced DM: chemically induced, insulin-dependent diabetes mellitus, ABA: abscisic acid, BW: body weight, BAT: brown adipose tissue, CAT: catalase, DM: diabetes mellitus, T2DM: type 2 diabetes mellitus, T1DM: type 1 diabetes mellitus, FFE: figs fruit extract, GLUT4: insulin regulated glucose transporter, FFA: free fatty acid, HDL: high density lipoprotein, HFD: high fat diet, ITT: insulin tolerance tests, Kidney Fxn: kidney function test, LDL: low density lipoprotein, MDA: malondialdehyde, OGTT: oral glucose tolerance, PPARγ: peroxisome proliferator-activated receptor gamma, STZ: streptozotocin, SOD: superoxide dismutase, TC: total cholesterol, TG: triglyceride, TNFα: tumor necrosis factor alpha, VLDL: very low density lipoprotein, WAT: white adipose tissue.

#### 2.4.3. Obesity, Satiety, and Dietary Patterns

Two articles were identified investigating the effect of fig fruit and fig leaf extract on body weight endpoints in rats (Table 4). The data indicates the anti-obesity activity of fig fruit when tested in a dose-response study design (100, 150, 200 mg/kg) including drug control ayurslim [117]. The leaf extract also induced weight loss in rodents [118]. In humans, however, no data examining body weight or satiety as a primary outcome variable was identified in the peer-reviewed literature. Bodyweight monitored as secondary or tertiary endpoints in other fig research revealed neutral results in humans [93] or decreased body weight in animal models of T2DM [109,110,113]. An assessment of changes in dietary patterns suggests fig intake (120 g/d, CA Mission) displaces other foods such as desserts, grains, dairy, and beverages when included in the diet for 5 weeks [119] (Table 5). NHANES data suggest the intake of dried fruits is associated with a lower body mass index and smaller waist circumference [120,121]. Overall, the benefits associated with fig consumption by humans, albeit limited, support further research into the satiety, body weight, and glycemic control of figs when included in the diet regularly.

**Table 4:** Obesity.

		Study Details		Intervention			Results
First Author Year	Study Design	Animals Used	Sample Size	Duration	Fig (Tx)	Control (Tx)	Endpoints
Animal Research							
Surendran, S, 2020 [117]	Parallel in vivo Animal	Male Swiss albino mice 25–30 g	3 groups	40 days	Fruit 100, 150, 200 mg/kg	Normal diet Cafeteria Diet Atherogenic diet	↓ BW
Noordam, E, 2019 [118]	Parallel in vivo Animal	high fat diet (HFD)-induced obese rat model	30 5/group	40 days	Leaf extract 100, 200, 400 mg/kg	Control	↓ BW @400 mg/kg

↓ (decrease). BW: body weight.

**Table 5:** Dietary Patterns.

			Study Detail		Results
First Author Year	Study Type Design	Subject Detail	Epi Type	Sample Size	Key Results
Human Research					
Sullivan, VK, 2021 [121]	Epi Cross Sec NHANES 2007–2016	US adults ≥20 y	Cross-sec	*n* = 25,590 1 diet record *n* = 22,311 2 diet record	Dried fruit consumers, *n* = 1233 dried fruit intake was 0.04 ± 0.001 cup-equivalents and represented 3.7% of total fruit consumed Consumers of dried fruit (7.2% of adults) had higher quality diets than non-consumers (mean ± standard error Healthy Eating Index 2015 score = 60.6 ± 0.5 vs. 52.6 ± 0.3; *p* < 0.001) and lower mean BMI, waist circumference, and systolic blood pressure (*p* < 0.01)
Alshaeri, HK, 2015 [119]	Human RCT crossover	56 y	*n*/A	88	Fig supplementation (120 g/d) on dietary patterns (vs. standard diet): ↑ Ca, ↑ K, ↑ Mg Figs displaced in diet: desserts ~4%, vegetables ~5%, dairy 10%, grain 23%, beverages 168% ↔ blood mineral status
Keast, D, 2011 [120]	Epi Cross Sec NHANES 1999–2004	US adults ≥19 years	Cross Sec	*n* = 13,292 1 diet record	~7% were dried fruit consumers Healthy Eating Index 2005 score 59.3 ± 0.5 vs. 49.4 ± 0.3 in consumers and non-consumers, respectively *p* < 0.05. Lower BMI, waist circumference, fewer short fall nutrients in consumers vs. non-consumers

Arrows: ↑ (increase), ↔ (no effect). BMI: body mass index, Ca: calcium, Epi-cross-sec: epidemiological cross section. RCT: randomized controlled trial, K: potassium, Mg: magnesium, NHANES: national health and nutrition examination surveys.

#### 2.4.4. Emerging Areas of Figs Health Benefits (Cognitive Function and Digestive/Gut Health)

Alzheimer’s disease (AD) is one of the most common forms of dementia in the elderly and is one of the most widely researched areas today. Excessive oxidants, such as reactive oxygen species (ROS) and inflammatory entities, are considered to be at the root of AD development. Figs are rich in fiber, a number of micronutrients including copper, iron, manganese, magnesium, potassium, calcium, and vitamin K, and an array of polyphenol compounds with demonstrated antioxidant and anti-inflammatory properties. Selected polyphenol metabolites cross the blood–brain barrier and may influence oxidative stress, inflammation and other signaling pathways important for disease prevention. During the last couple of decades, fruits, particularly berries, have been investigated for their effects on cognitive function. Figs, mainly dark figs, share some of the same types of anthocyanins as berries that may have effects on the brain. Subash et al. (2016) published on fig fruits grown in Oman, showing that dietary supplementation with 4% figs protected against memory decline, increased anxiety-related behavior, and reduced severe impairment in spatial, position discrimination learning ability, and motor coordination in APPsw/Tg2576 (Tg mice) mice, a standard rodent model for AD. The authors concluded that dietary supplementation of figs may be useful for improvement in cognitive and behavioral deficits in AD [122] (Table 6).

Figs have been traditionally used for improving digestive health. Only two studies have been conducted so far to examine the impact of figs on digestive/gut health, one involving humans and the other animals. In a randomized control trial conducted in humans with irritable bowel syndrome with predominant-constipation (IBS-C), dried figs (45 g) or dried flixweed (30 g) were given to patients. The results showed a substantial improvement in IBS-symptoms including a reduction in the frequency of pain, defecation, and hard stool after intake of figs or flixweed compared to the control [123]. In an animal study, the ameliorative effect of *Ficus carica* L. aqueous extracts (FCAE) was studied in DSS-induced colitis rats. The FCAE was administered orally to the rats at a dose of 150–300 mg/kg once a day for 7 days. The oral intake of FCAE significantly increased gastrointestinal transit-ratio and gastric-emptying by hastening their times, and reduced the constipation severity which was induced by the colitis [124] (Table 6). These findings provide a foundation for future research on the therapeutic properties of figs in improving digestive health.

**Table 6:** Cognitive function and gut/digestive health.

		Study Details		Intervention			Results
First Author Year	Study Design	Humans/Animals	Sample Size	Duration Fig Control			Various End Points
Cognitive Function							
Animal Research							
Subash, S, 2016 [122]	in vivo animal parallel AD model of disease	APPsw/Tg2576 (Tg mice) mice model for AD vs wild type	12 Tg mice 6 wild mice (control, non-Tg)	15 months	4% of diet	w/o Fig	Fig prevented memory decline in Tg mice Fig prevented declines in spatial, position discrimination learning ability, and motor coordination ↓ anxiety
Gut/digestive health							
Human Research							
Pourmasoumi, M, 2019 [123]	RCT	Adults with IBS	150	4 months	Fig vs. Flixweed	Control	Flixweed or FIG vs. control: ↓ IBS symptoms ↓ frequency of pain, ↓ distention ↓ frequency of defecation ↓ hard stool. ↑ QOL ↑ satisfaction w/bowel habits. ↔ abdominal pain severity ↔ C-reactive protein
Animal Research							
Rtibi, K, 2018 [124]	Parallel in vivo animal	Colitis model DSS-induced UC rat model	Not mentioned in paper	7 days	Fig extract 150–300 mg/kg	Control	Improved management of several colitis induced endpoints: AOX fecal water content lipid metabolism gastric emptying and GI motility

Arrows: ↑ (increase), ↓ (decrease), ↔ (no effect). AD: Alzheimer’s disease, AOX: antioxidant activity, APPsw: microinjected mice express a mutated form of human gene for amyloid precursor protein (APP) known as Swedish mutation, BMI: body mass index, DSS induced UC: dextran-sulfate-sodium-induced ulcerative colitis, GI: gastrointestinal, IBS: irritable bowel syndrome, QOL: quality of life, RCT: randomized controlled trial, Tg, transgenic.

## 3. Potential Mechanisms Involved in Health Benefits of Figs

Reactive oxygen and nitrogen species (ROS/RNS) are continuously formed during normal metabolic reactions. However, under normal physiological conditions, their levels are regulated by antioxidant defense systems by both enzymatic and non-enzymatic pathways, operating in intracellular and extracellular spaces, preventing or delaying oxidative damage of cellular compounds [125]. In chronic disease conditions such as obesity, diabetes, and cardiovascular diseases, ROS/RNS are produced excessively, making the redox state of cells shift towards oxidizing conditions and cause inflammation [126]. Figs contain polyphenolic compounds that have been shown to exert antioxidant activity in in vitro systems [5]. However, clinical studies are not available to show the direct antioxidant activity of the figs in biological systems, but their downstream effects may be apparent in clinical outcomes. The cardiometabolic benefits of plant polyphenolic compounds are proposed to be mediated, at least in part, through redox-sensitive cellular signaling pathways that reduce oxidative stress and inflammation.

## 4. Summary/Conclusions/Future Research

The phytochemistry of figs grown in different parts of the world other than the USA is well represented; however, limited information is available about phytochemical composition of fig varieties (dried and fresh) grown in the USA. From the available literature on figs, anthocyanins, rutin, and carotenoids are the primary phytochemical classes represented in figs, though other flavonoids and phenolic compounds are also present. Darker varieties and fresh/unprocessed figs tend to have higher densities of select phytochemicals; however, growing region, variety, harvest time, and agronomic practices all play a role in phytochemical composition and content. An evaluation of the literature characterizing the bioavailability of fig nutrients and phytochemicals revealed limited data. Future studies focused on understanding the bioavailability of phytochemicals in figs after they are consumed by humans, both in terms of short-term (one-time intake) and long-term (regular intake for a month or more) interventions will reveal new information about figs and how they can be applied in the diet to promote specific health objectives. The gut microbiome is also an area of interest, as changes in the composition and function of the gut microbiota may affect the bioavailability of the phytochemicals in figs. Research in animal and human models of health and disease have tested the biological activity of the fruit/pulp, peels, and leaf extracts consumed over days to weeks. Despite the promising preliminary research of figs and extracts from fig parts, additional well-controlled human studies, particularly using fig fruit, will be required to uncover and verify the potential impact of dietary intake of figs or nutraceutical applications on critical health issues such as managing cardiovascular disease, diabetes, and supporting gut health. Other areas such as satiety and cognitive function may also be worthy of exploration as evidence develops.

## Figures and Tables

**Table 1:** Keywords used to search fig literature in Medline with PubMed.

Figs ^1^	Dried Figs ^1^	Fresh Figs ^1^
Appetite	Alzheimer’s disease	Absorption
Blood pressure	Body weight	Bioavailability
Cardiovascular disease	Chemistry	Cholesterol
Cognition	Diabetes	Food intake
Glucose	Gut health	Heart disease
Insulin	Insulin resistance	Lipids
LDL cholesterol	Microbiome	Metabolism
Nutrients	Obesity	Phytochemicals
Polyphenols	Type 2 diabetes	

^1^ Searched alone and in combination with other keywords, such as “dried figs and appetite”.

## Data Availability

Not applicable.

## Supplementary Materials

The following are available online at https://www.mdpi.com/article/10.3390/nu15112623/s1, Table S1: Phytochemicals in Figs.
