# Supplementary material for: Phytochemical Composition and Health Benefits of Figs (Fresh and Dried): A Review of Literature from 2000 to 2022
Source: Nutrients. 2023 Jun 3;15(11):2623. doi: 10.3390/nu15112623 (PMC10255635; doi:10.3390/nu15112623)
Supplement: Supplementary file 1 [file nutrients-15-02623-s001.zip › nutrients-2399156-supplementary.pdf]

Phytochemical Composition and Health Benefits of Figs (Fresh and Dried): A Review of Literature from 2000 to 2022

Amandeep K. Sandhu, Maria Islam, Indika Edirisinghe and Britt Burton-Freeman *

Department of Food Science and Nutrition, Center for Nutrition Research, Institute for Food Safety and Health, Illinois Institute of Technology, Chicago, IL 60616, USA; asandhu2@iit.edu (A.K.S.); mislam17@hawk.iit.edu (M.I.); iedirisi@iit.edu (I.E.)

* Correspondance : bburton@iit.edu; Tel.: +1-708-341-7078

Supplementary Table S1: Phytochemicals in Figs

First Author<br>Year	Study type	Study methods	Fig type/varieties	Geographical location	Major findings
Hoxha L<br>2022 [68]	Harvesting effect	PCs: TPC, TFC, TAC<br>AOX: ABTS<br>Physico-chemical parameters	2 fig varieties<br>Black and white	Albania	TPC and AOX decrease continuously during fruit development and ripening with 1.91-fold<br>and 2.45-fold decreases observed in the second and third stage of maturation, respectively, compared to the first one.<br>Black varieties had highest TPC and AOX
Karantzi AD<br>2021 [48]	PCs at different ripening stages	Color<br>PCs: anthocyanins and phenolic acids by HPLC-DAD<br>AOX: FRAP<br>Total chlorophyll and carotenoids	3 fig varieties<br>Four ripening stages<br>Pulp and peel	Greece	Gallic acid was the prominent acid in flesh and cyanidin-*O*-3-rutinoside the major anthocyanin in pulp and peel in all varieties<br>2,4-dihydroxybenzoic (2,4-DHB), 2,3-dihydroxybenzoic (2,3-DHB) and sinapic acids, and the anthocyanins delphinidin-3-*O*-glucoside, petunidin-3-*O*-glucoside in flesh and malvidin-*O*-glucoside in pulp and peel were identified for the first time in figs<br>PCs, carotenoids and AOX didn't change with ripening in pulp but significant increase in anthocyanins was observed in peel of all the varieties

Tewari D<br><br>2021 [60]	Extraction comparison using various techniques	Extraction: methanol, boiling water and soxhlet using methanol<br><br>PCs: TPC, TFC<br><br>UHPLC-ESI-MS<br><br>AOX: DPPH, FRAP, ABTS, CUPRAC, phosphomolybdenum, and chelating tests<br><br>Enzyme inhibitory activity	Wild Himalayan fig (*F. palmata* Forssk.)	India	TPC: water extraction >Soxhlet> methanol<br><br>TFC: methanol>Soxhlet>water extraction<br><br>Highest number of compounds identified in methanol extract (43) followed by Soxhlet (41) and water extracts (37)<br><br>DPPH and ABTS radical scavenging highest in aqueous extract<br><br>Methanol extracts had better enzyme inhibitory activity compared to water extract
Paramanandam V<br><br>2021 [74]	Drying effect	Drying methods: sun drying (SD), hot-air drying (HD) and microwave drying (MD)<br><br>Proximate composition<br><br>PCs: TPC, TFC, TTC<br><br>HPLC-DAD<br><br>AOX: FRAP, DPPH, ABTS	Roxburgh fig (*Ficus auriculata* Lour.)	India	After HD:<br><br>↑ carbohydrates<br><br>↑ proteins<br><br>↑ amino acids<br><br>↑ gallic acid<br><br>After MD:<br><br>↑ Caffeic acid, rutin<br><br>↑ AOX<br><br>↑ Nutritional value

		Scanning electron microscope (SEM) imaging			SEM: MD showed a loser porous structure, good for preservation
Hssaini L<br><br>2021 [16]	Variety comparison	Fruit peel color<br><br>PCs: TPC<br><br>AOX: ABTS, DPPH, FRAP<br><br>Sugar and organic acid by HPLC<br><br>FTIR fingerprinting	11 fig varieties<br><br>Lyophilized figs used in powder form.	Morocco	FTIR analysis displayed six major fingerprints<br><br>Significant variability between fruit peel color, sugars, organic acids, AOX and TPC among the 11 varieties ($p < 0.001$)<br><br>Malic acid was the most abundant organic acid in all samples<br><br>AOX by DPPH>FRAP
Hssaini L<br><br>2021 [42]	Chemical and chemometric approach to fig peels and pulp	Color by colorimeter<br><br>PCs: TPC, TAC, TFC, TPAC<br><br>HPLC-DAD<br><br>AOX: ABTS, DPPH	25 Fig varieties<br><br>Peel and pulp	Morocco	The fig peels and pulps color showed significant differences among varieties at<br><br>$p < 0.001$, with the exception of the pulp lightness coordinate (L*)<br><br>Significant variation in TPC, TAC, TFC, TPAC among various varieties.<br><br>PCs > 2 times higher in peel than pulp<br><br>AOX: Peel > pulp<br><br>HPLC-DAD: 12 compounds identified in peel and 8 in pulp of various varieties

Lachtar D<br><br>2021 [75]	Drying effects on figs quality	Drying methods: open air solar drying and greenhouse solar drying<br>Fruit color<br><br>Mineral content by atomic absorption spectroscopy<br><br>PCs: TPC<br>AOX: DPPH	2 fig varieties	Tunisia	Green house drying improved fig color<br><br>Green house drying ↑ TPC and AOX, color and ↓ trace elements in both varieties
Gündeşli M<br><br>2021 [24]	Polyphenolic composition at 4 different harvesting time periods	PCs: TPC, TFC, TAC<br><br>HPLC-DAD<br><br>AOX: DPPH	Local fig variety	Turkey	14 different phenolic compounds characterized at different harvest periods.<br><br>Epicatechin (7.809 mg/100 g) was dominant.<br><br>TPC, TFC, TAC and AOX were highest at<br><br>the 1st harvest and lowest at 4^{rth} harvest
Aljane F<br><br>2020 [32]	Phytochemicals and AOX	PCs: TPC, TAC, TFC<br><br>AOX: ABTS and DPPH<br><br>Reducing sugars: HPLC-RID	27 fig varieties<br><br>Green-yellowish<br><br>Green<br><br>Red-greenish<br><br>Brown-purplish	Tunisia	Significant variation in the PCs, AOX and sugar<br><br>compositions (glucose and fructose) in the 27 Tunisian fig varieties.<br><br>Purplish black varieties had the highest TAC.

			Purple-greenish<br>Purple-blackish		
Hssaini L<br>2020 [34]	Morphological assessments<br>Phenolic composition<br>Variety comparison	Morphological traits<br>organic acids, sugars<br>PCs: TPC, TFC, TAC AOX: DPPH, FRAP and ABTS<br>color analysis	11 fig varieties	Morocco	The variety 'Kadota' had the most promising morphological traits<br>Reducing sugars were same in all varieties<br>Malic acid predominant organic acid in all varieties<br>TPC, TFC and TAC highest in dark-colored varieties<br>TPAC highest in light skinned varieties.<br>DPPH and ABTS higher in light-colored figs,<br>while, FRAP was generally higher in dark skin-colored varieties.
Ara I<br>2020 [37]	PCs<br>AOX<br>Antidiabetic activity	AOX: DPPH<br>PCs: HPLC-DAD<br>Anti-diabetic activities (α-amylase inhibition)	1 fig variety<br>pulp, peel and leaves	Pakistan	Leaves>peel>pulp in TPC, AOX and α amylase inhibition
Zhang<br>2020 [56]	Quality and morphology at different ripening stages	Morphological indices<br>Color and taste<br>Anthocyanins and organic acids: HPLC	6 fig varieties<br>Yellow<br>Red<br>Purple	China	During the ripening of fig, malic acid, quinic acid, and citric acid were the main organic acids, followed by tartaric acid and succinic acid.<br>Fumaric acid present in lowest content.

			Peel and pulp		The highest anthocyanin content was observed for cyanidin-3-*O*-glucoside, followed by cyanidin-3-*O*-rutinoside, and cyanidin-3,5-diglucoside had the least content.<br><br>There was change in accumulation pattern of organic acids at different ripening stages however, no changes observed in anthocyanins.
Petkova N<br><br>2019 [76]	Changes in phytochemicals	Physicochemical parameters<br><br>PCs: TCC, TPC, TFC, TAC<br><br>AOX: DPPH, FRAP<br><br>Sugars: HPLC-RID	1 yellow-green color fig variety<br><br>fresh<br><br>frozen<br><br>jam	Bulgaria	The highest values of PCs, sugars and AOX were found in fresh fruits, while in frozen fruits their levels decreased significantly.<br><br>The preparation of fig jam was a better approach for preserving some bioactive compounds, especially carotenoids and PCs.
Hssaini L<br><br>2019 [35]	Physio-biochemical screening	PCs: TPC, TFC, TAC, TPAC<br><br>Physicochemical parameters<br><br>AOX: DPPH, ABTS and β carotene blanching test	135 varieties 94 local clones & 41 imported varieties	Morocco	All analyses revealed significant variation among varieties<br><br>Dark fig varieties highest TPC, TAC and AOX
Palmeira L<br><br>2019 [40]	Characterization of nutrients including phytochemicals	Nutritional and chemical composition<br><br>sugar analysis: HPLC-RID, organic	1 fig variety<br><br>lyophilized ("Pingo de Mel")<br><br>Peel and pulp	Portugal	Peel sample presented significantly higher contents of ash, fat and carbohydrates than the pulp<br><br>Glucose, fructose, sucrose and threalose detected in both peel and pulp

		acids by HPLC-PDA, tocopherols by HPLC-fluorescence detector, fatty acids by GC-FID<br><br>AOX: DPPH, reducing power, β carotene bleaching assays,<br><br>TBARS, and the oxidative haemolysis inhibition<br><br>assay (OxHLIA)<br><br>PCs: LC-DAD-ESI/MS^n<br><br>Antibacterial activity			5 organic acids were identified and quantified namely oxalic, quinic, malic, citric, and succinic acids.<br><br>Tocopherols detected in all their 4 forms<br><br>23 fatty acids detected in samples<br><br>12 & 15PCs in pulp and peel, respectively.<br><br>Rutin content highest in peel<br><br>The peel presented significantly lower IC50 values than pulp<br><br>Fig peel is superior to the corresponding pulp in terms of nutritional and phenolic profiles as well as bioactivities
Khadhraoui M<br><br>2019 [17]	Phytochemical composition	PCs: TPC, TFC<br><br>LC-MS and GC-MS<br><br>AOX: ABTS, DPPH	9 sun-dried varieties<br><br>Skin color varied<br><br>Black<br><br>Green-yellow<br><br>green	Tunisia	Significant variations among the varieties in all analyses<br><br>Dark fruits: higher TPC (~2.5 times higher) than green fruits<br><br>Fatty acids (FA): palmitic, oleic, linoleic, linolenic and trace amounts of arachidic acid

			purple		
Mahmoudi S<br><br>2018 [41]	Proximate composition, phenolic composition, antioxidant capacity<br><br>Consumer acceptance	Pomological assessment, skin color, nutritional analysis, consumer acceptance test<br><br>PCs: TPC, TAC, TFC, CT (condensed tannin)<br><br>AOX: DPPH	9 fresh fig varieties<br><br>Peel and pulp	Algeria	Skin color results indicate two groups (light skin and dark skin groups)<br><br>Organic acids in fig pulp > than peel<br><br>K and Ca two major minerals present in different varieties<br><br>Peel> than pulp in TPC, TAC, TFC, CT and AOX<br><br>Consumers preferred less sweet, juicier and easier to peel varieties
Meziant L<br><br>2018 [62]	Extraction efficiency	TAC<br><br>extraction variables: solvent, #extractions,<br><br>solid-to-solvent ratio, extraction time, methanol conc., type of acidification, and acid-to-solvent ratio (1/99 to 20/80).	1 fig variety<br><br>peel	Algeria	Optimum extraction conditions for Anthocyanins:<br><br>Double extraction with 90% methanol acidified to a ratio of 10/90 with 5% citric acid using a solid-to-solvent ratio of 1/100 extraction time, 180 min<br><br>The extract yielded 346.63 mg monomeric anthocyanins per 100 g of dried fig peels.
Sedaghat S<br><br>2018 [83]	Enzyme activity, Sugar and organic acids	Chemical composition<br><br>Sugars and organic acids: HPLC	1 fig variety with samples collected at 4 different stages over 2	Iran	Glucose and fructose significantly increased as fruits developed.<br><br>Citric acid was the most abundant organic acid followed by malic acid.

		Enzyme activity assays	consecutive years		Invertase activity increased sharply before the caprification stage up to the senescence stage.<br><br>Fig fruit possess α- and β-amylase activities, which increased up to the ripening stage and then slightly decreased at the senescence stage.
Sedaghat S<br><br>2018 [70]	Physicochemical changes in fruit development	Color analysis, mineral analysis, crude fiber, aflatoxin<br><br>PCs: TPC<br><br>HPLC-DAD	1 fig variety with samples collected at 4 different stages over 2 consecutive years	Iran	K and Fe high in dried fig on trees<br><br>Total PCs decreased until fruit ripening, followed by an increase at the stage of senescence<br><br>(+) Catechin, Chlorogenic acid, (−) Epicatechin and Quercetin-3-*O*- glucoside highest when the fruits dried on the trees.<br><br>% crude fiber stable until development
Mopuri R<br><br>2018 [39]	Phytochemical extracts of figs<br><br>metabolic syndrome	PCs: TPC, TFC<br><br>AOX: DPPH<br><br>anti-diabetic and anti-obesogenic activity by enzymatic assays<br><br>GC-MS	leaves and stembark from India<br><br>Fruit from S. Africa	India<br><br>South Africa	Fruit extract significantly ($p < 0.05$) > than all other extracts and parts of the plant in terms of AOX, antidiabetic, and antiobesogenic effects<br><br>GC-MS (13 components detected)

Backes E<br><br>2018 [61]	Extraction efficiency	solid-liquid extraction methods were optimized by response surface methodology<br><br>selected techniques: heat extraction (HE), ultrasound assisted extraction (UAE), microwave extraction (ME)<br><br>Anthocyanin analysis: HPLC-DAD-ESI/MS	Lyophilized peel powder from 1 local variety	Portugal	UAE of anthocyanins > HE and ME<br><br>UAE was the most effective method, yielding 3.82 mg C3R per g of the extracted residue at the optimal global extraction conditions (21 min, 310 W, and 100% of ethanol).
Maghsoudlou E<br><br>2017 [18]	Extraction and AOX activity	Sub-critical water extraction<br><br>PCs: TPC, TFC<br><br>AOX: DPPH, reducing power, rancimat tests<br><br>Antioxidant potential in canola oil	2 fig varieties<br><br>Pulp and skin<br><br>dark purple<br><br>green	Iran	AOX of dark variety extract > green extract; AOX of skin extracts > than pulp extracts.<br><br>Dark fig skin extracts comparable to the synthetic antioxidant (TBHQ) for oxidative stability in canola oil
Pereira C<br><br>2017 [82]	Physico-chemical and	Nutrients & quality analysis at different	9 fig varieties	Spain	Early ripening stage ↑ (fruit size, fiber, protein, TA of some varieties)

	nutritional characterization	ripening stages<br>Organic acids			Later ripening ↑ (TSS, MI, color intensity.)<br>↔ organic acids between varieties and ripening stages
Ersoy N<br>2017 [19]	Physico-chemical properties	Various physico-chemical properties<br>AOX: DPPH, Fe^{2+} chelating and H_2O_2 scavenging activity and metal chelating<br>PCs: TPC	4 fig varieties<br>Black<br>Purple<br>Green<br>yellow	Turkey	Significant differences in physico-chemical properties, AOX and TPC among 4 varieties<br>Varieties with dark colors had higher AOX and PCs than those with lighter color.
Konak R<br>2017 [20]	Drying method effects	Oven vs sun drying<br>AOX: TEAC, ABTS<br>PCs: TPC	4 fig varieties<br>2 dark-colored (black) and 2 light-colored (yellow)	Turkey	TPC, AOX of fresh dark figs > fresh light figs<br>TPC of dried dark figs > light figs<br>However, AOX of dried dark figs < light figs<br>No effect of sun or oven drying on TPC, AOX in 3 out 4 fig varieties
Wang Z<br>2017 [71]	Metabolomic and transcriptomic analyses	HPLC-ESI-QQQ/MS/MS<br>RNA sequencing<br>Real time quantitative PCR	2 fig varieties	China	5 and 22 metabolites were identified as having significantly different contents between fruit peels of the two varieties at young and mature stages, respectively.

					Significant variation in accumulation of various flavonoids including anthocyanins at young and mature stages.<br>Upregulation and down regulation of genes involved in flavonoid biosynthesis pathway based on maturity stages
Bey MB<br>2016 [73]	Sun drying effects	Physico-chemical parameters<br>AOX: ABTS, ferric reducing power, β-carotene-linoleic acid assay<br>PCs: HPLC-DAD	3 dark fig varieties	Algeria	22 PCs identified<br>Sun drying<br>↑ sugar content<br>↓ PCs<br>↓ AOX<br>↓ Flavonoids by 86% with complete loss of C3G and C3R
Pourghayoumi M<br>2016 [15]	Comparison of varieties phenolic content and composition	PCs: TPC, TFC, TAC<br>HPLC-UV/vis<br>AOX: DPPH<br>TSS, color parameters	9 dried fig varieties	Iran	Significant differences were determined among the varieties with respect to the distribution of PCs<br>AOX: no significant differences observed except for one variety<br>TAC negligible in all varieties
Ajmal M<br>2016 [36]	Nutritional characterization	Proximate analysis<br>PCs: TPC, TFC, TAC	1 fig variety<br>Black	Pakistan	PCs in figs leaves>peel>pulp<br>C3R highest in peel extract

	hypoglycemic potential	HPLC for cyanidine-3-rutinoside (C3R)<br>AOX: FRAP, DPPH<br>Hypoglycemic potential (Rat study for 56 days)	leaves, peel and pulp		Leaf extracts highest AOX and alleviates the glucose and insulin levels in rats
Wojdyło A<br>2016 [44]	Comparison of anti-diabetic and AOX of different varieties of figs	antidiabetic effects<br>polyphenolic and triterpenic composition: LC-MS QTOF<br>AOX: ORAC<br>sugar composition	10 different varieties with 2<br>types (brevas and figs)	Spain	Phenolic compounds varied significantly by variety<br>11 PCs were identified<br>Betulinic acid was the main triterpenoid followed by oleanolic acid.<br>ORAC/AOX- highest in Verdal brevas<br>Anti-diabetic enzyme inhibition varied with varieties
Harzallah A<br>2016 [21]	Phytochemical content and AOX of juices from different parts of fig	PCs: TPC, TFC, total ortho-diphenols content, TAC, TTC<br>AOX: DPPH, reducing power assay	3 Fig varieties<br>Green, purple, black<br>Juices of peel, pulp and whole fruit	Tunisia	Black peel and fruit juice highest AOX<br>With ripening ↑ AOX ↑ PCs
Hoxha L<br>2016 [31]	PCs and AOX<br>Breba and main crop	PCs: TPC, TAC, TFC<br>AOX: DPPH, ABTS	2 fresh fig varieties<br>Purple<br>Green	Albania	The main crop of both varieties had higher TPC compared to breba crop.<br>Dark variety and peel had higher AOX, TFC and TPC

			Whole fruit, pulp and peel		
Hoxha L<br>2015 [22]	AOX of dried autochthonous figs	PCs: TPC, TAC, TFC<br>AOX: DPPH, ABTS	5 Fig varieties<br>Yellow, green, brown, purple, black	Albania	TPC, TAC, AOX, TFC higher in dark compared to light fig varieties.
Bey MB<br>2015 [23]	Phytochemical content and AOX of dried dark and light fig varieties	PCs: TPC, TAC, TFC, TPAC and flavanol content<br>AOX-DPPH, H_2O_2 and O2 scavenging effects phosphomolybdenum assays	9 dried Fig varieties<br>3 dark<br>6 light	Algeria	Dark fruits had higher PCs, AOX compared to light fruits<br>High correlation between AOX and phytochemical assays
Feng YC<br>2015 [58]	Purification of phenolic compounds	total sugars<br>PCs: TPC<br>AOX: ABTS, FRAP<br>Purification parameters optimization,<br>MEKC	1 fig variety	China	Increased AOX, catechin, epicatechin, chlorogenic acid, and rutin after purification compared to crude extract

Ammar S<br>2015 [49]	Phenolic compound and AOX	AOX: TEAC, FRAP, ORAC<br>PCs: TPC<br>UHPLC-DAD-MS	2 fig varieties<br>Black and green<br>leaves, fruits, skins and pulps	Tunisia	116 compounds characterized in different parts<br>Leaves of both green and dark high in PCs<br>Rutin- main component in fruits, skin & leaves<br>prenylhydroxygenistein-major component in pulp<br>9 anthocyanins characterized: cyanidin 3-rutinoside and cyanidin 3,5-diglucoside major ones<br>Good correlation between (AOX, TP, PCs)
Kamiloglu S<br>2015 [52]	Effect of sun drying on PCs<br>Bio-accessability	PCs: TPC, TFC, TPAC, TAC<br>HPLC-PDA<br>AOX: DPPH, ABTS, FRAP, cupric ion reducing antioxidant capacity (CUPRAC)<br>*In vitro* GI digestion	2 fig varieties<br>Yellow and purple	Turkey	14 phenolic compounds were analyzed<br>Rutin and cyanidin-3-rutinoside were major flavonol and anthocyanin, in both varieties, respectively<br>Yellow figs: higher TFC and TPAC<br>Sun drying ↓ TPC, TAC, AOX<br>Bio-accessability of PCs: high for skin of both varieties; ↓ after sun drying in pulp
Qin H<br>2015 [63]	Ultrasound assisted extraction (UAE)	Ionic liquid-based UAE<br>Optimization parameters: extraction solvent concentration, solid/liquid ratio, extraction time and temp	The leaves, pulps, and peels of fig<br>Variety not provided	China	UAE was used to extract gallic acid, chlorogenic acid, rutin, psoralen, and bergapten from different parts of fig fruit.<br>Maximum extraction of PCs was achieved with these optimal conditions: e.g., with 1.0 M<br>[BMIM][PF6], solid–liquid ratio of 1:50, ultrasound extraction time of 30 min and extraction temperature of 30 °C

		HPLC-UV			
Trad M<br>2014 [55]	Nutritive and polyphenol content comparison	Sugars, organic acids and alcohol insoluble solids<br>PCs: HPLC-DAD	5 fig varieties<br>2 dark<br>3 yellow green	Tunisia	Glucose and fructose major sugars<br>Citric acid major organic acid in all varieties<br>Cyanidin-3-rutinoside was the most abundant compound among all varieties
Debib A<br>2014 [65]	AOX and antimicrobial activity<br>Extraction	Extraction: methanol, water, petroleum ether and acetone<br>Antimicrobial activity<br>AOX-DPPH<br>PCs: TPC, TFC & tannin content	2 dried fig varieties	Algeria	High AOX of methanol extract of Ajendar variety<br>High Antimicrobial activity of methanolic extract of Taamriout variety<br>High TPC and TFC in acetone and aqueous extracts
Jokić S<br>2014 [66]	Comparison of PCs extraction	PCs: TPC, TFC<br>Color analysis<br>Ultrasound assisted Extraction (UAE)<br>Solid-liquid extraction (SLE)	5 dried fig varieties	Croatia	TPC&TFC: UAE>SLE<br>Significant differences in TPC and TFC in 5 different fig varieties<br>Color changes in extracts ↓ by UAE<br>The darker varieties had highest TPC and TFC
Russo F<br>2014 [45]	PCs comparison in different varieties, fresh vs dried,	TPC<br>HPLC-UV/DAD	19 fig samples	Italy<br>Turkey<br>Greece	Fig peel high in PCs<br>Significant qual and quant differences in PCs among fresh/dried fig samples from different origins

	different regions and breba vs full crop		9 fresh "Dottato" fig samples<br>from Italy, 10 dried fig samples from Italy (2), Turkey (6), and Greece (2)		First crop richer in PCs than the 2nd crop.<br>Turkish dried figs had the highest amount of PCs
Sadia H<br>2014 [79]	Nutrient and mineral content	Proximate composition<br>Mineral analysis by AAS.	3 fig varieties	Pakistan	Significant variation existed among the selected fig varieties in all the nutritional parameters<br>Na, K and Mg major minerals in figs
Soni N<br>2014 [86]	Nutritional, phytochemical, AOX and antibacterial activity	Nutritional content<br>PCs: TPC, TFC, crude alkaloids and saponins<br>GC-MS screening of volatile and semi-volatile compounds<br>AOX: FRAP, ABTS<br>Antibacterial activity	Dried figs purchased from local market	India	Dried fig good source of minerals like Sr,<br>Ca, Mg, P and Fe.<br>vitamin E, β-amyrin, stigmasterol, campesterol, oleic acid, isoamyl laurate and ϒ tocopherols were identified with GC-MS

Tanwar B<br><br>2014 [77]	Processing into fig jam and nectar	Nutritional analysis<br><br>Physicochemical analysis<br><br>PCs: TPC, TFC, TAC, TTC	Figs purchased from local market	India	Processing of fig fruit pulp into jam and nectar resulted in a significant ($p<0.05$) increase in physicochemical properties like TSS and TA, but a significant ($p<0.05$) decrease in pH, iron, calcium and phosphorus; TPC, TFC, TAC, TTC, beta carotene, crude fiber, protein, fat, and vitamin C
Bey MB<br><br>2014 [67]	Optimization of recovery of PCs and AOX	Response surface methodology<br><br>3 variables: solvent concentration, temperature and time<br><br>TPC and AOX by DPPH	1 dried dark fig variety	Algeria	The optimal extraction parameters were 61.03% acetone, 105.12 min, and 46.16°C.
Bey MB<br><br>2013 [64]	Extraction optimization and efficiency	solvents (acetone, ethanol, methanol and water) comparison for the extraction of PCs.<br><br>Extraction parameters: solvent type, solvent concentration, acid concentration, extraction time and temp, sample/solvent	2 fig varieties<br><br>Light (green)<br><br>Dark (black)	Algeria	All extraction parameters<br><br>had significant effects ($p<0.05$) on the TPC and AOX<br><br><br>Optimized conditions: double extraction using 60% acetone without acidification, at 40°C for 120 min, and with a 1/75 solid to solvent ratio

		ratio and no. of extractions<br>TPC & DPPH-test conducted to determine the extraction efficiencies			
Kamiloglu S<br>2013 [89]	Bio- accessibility of PCs.	*in vitro*-simulated GI tract digestion<br>AOX: DPPH, FRAP, ABTS<br>PCs: TPAC<br>HPLC-PDA	2 fig varieties<br>Purple<br>Yellow<br>skin, pulp, whole-fresh and<br>whole-dried fruit	Turkey	Sun-drying of fig fruit might result in an ↑ bio-accessibility of TPAC and chlorogenic acid content as well as AOX.<br>Anthocyanin bio-accessibility ↓ as a result of sun-drying.
Nakilcioğlu E<br>2013 [46]	PCs in Sarilop fig type	PCs: TPC, TFC<br>HPLC-DAD<br>AOX: DPPH, FRAP	10 fig varieties<br>Fresh and dried	Turkey	PCs in fresh fig >dried figs<br>major compound: (-)-epicatechin<br>Significant differences in different fig varieties from different regions of Turkey
Vemmos S<br>2013 [85]	Seasonal changes in carbohydrate content	Carbohydrate analysis: HPLC-RID	3 fig varieties<br>Fruit, leaves	Greece	The main sugars found in leaves & fruits of the three varieties were sucrose, glucose and fructose<br>Compared to other sugars sucrose> in leaves while< in fruits

					At fruit maturation concentration of sugars ↑
Ercisli S<br>2012 [33]	Color<br>AOX	PCs: TPC, TAC<br>AOX: TEAC, FRAP<br>Color analysis<br>TTA and SSC	A total of 24 local fig<br>genotypes and two standard fig varieties<br>(Sarilop and Bursa Siyahi)<br>Light and dark colors	Turkey	Fruit skin color of genotypes were found to be very diverse, i.e., light green, light purple, purple, dark purple and black.<br>Lot of variation in fig genotypes based on studied parameters<br>Black/dark colored fig genotypes high in TPC, TAC and AOX<br>Local fig genotypes had higher AOX than other genotypes
Ouchemoukh S<br>2012 [43]	AOX activity	AOX: DPPH, reducing power & phosphomolybdenum method<br>PCs: TPC, TAC, TFC, TC, TPAC	Black fig variety along with other dried fruits	Algeria	Apricots & figs high in carotenoids<br>Figs had the highest concentration of TPC &TFC compared to other dried fruits
Mujic I<br>2012 [51]	AOX properties	Freeze dried fig extracts-70% methanol and 70% ethanol-compared<br>PCs: TPC, TFC<br>HPLC-MS<br>AOX: DPPH	5 fig varieties	Croatia	Methanol extracts better for TPC<br>Rutin was detected in all five samples, with HPLC/MS

Vallejo F<br>2012 [12]	PCs in dried &fresh figs	PCs: TPC,<br>LC–UV-DAD/ESI-MS^n	18 fresh fig varieties and 3 dried fig varieties<br>Dark and green varieties<br>Peel and pulp	Spain	PCs in 1st crop>2nd crop<br>PCs>in skin of fresh figs compared to pulp<br>Skin: mainly anthocyanins<br>pulp: mainly proanthocyanidins<br>C-glycosides were detected for the first time
Faleh E<br>2012 [50]	Phenolic profile variability	AOX: DPPH, NO and O_2^- scavenging activity<br>HPLC-DAD	17 dried fig varieties<br>green, red and black	Tunisia	quercetin-3-O-rutinoside was the major compound quantified.<br>All fig varieties exhibited AOX against DPPH and O_2^- radical in a concentration dependent way<br>Only “Hammouri” variety presented some capacity to scavenge NO radical.
Yemiş O<br>2012 [54]	Pigment profile and surface color	Anthocyanin & Carotenoid composition<br>Sun drying<br>Color: hunter lab system<br>HPLC/DAD & HPLC/MS	5 fig varieties<br>Green<br>Yellow<br>Purple	Turkey	cyanidin-3-rutinoside (major anthocyanin)<br>In yellow fig varieties; lutein, zeaxanthin, β-cryptoxanthin and β-carotene major carotenoids<br>~ 80% of carotenoid compounds in yellow fig varieties degraded at the end of drying<br>Browning of figs observed upon sun-drying
Bucić-Kojić A<br>2011 [59]	Extraction conditions effects on PCs	Diff. aqueous ethanol concentrations, extraction temp	5 fig varieties	Croatia	Best extraction conditions: (80%, v/v aqueous ethanol, 80°C)<br>The highest TPC was found in Crnica fig variety while the lowest in fig variety Bjelica.

		PCs: TPC, TFC, TPAC<br>AOX: DPPH			
Marrelli M<br>2012 [69]	Changes in PCs and lipophilic composition at different harvesting stages	PCs: TPC<br>GC-MS<br>free radical scavenging activity, β-carotene bleaching assay, photodynamic cytotoxicity & pancreatic lipase activity	1 fig variety	Italy	Fruits of the first harvest furanocoumarins pyranocoumarins while fruits in September highest TPC.<br>antiradical & lipid peroxidation activity exhibited by fruits from first harvest<br>Dichloromethane fractions highest photodynamic cytotoxicity
Slatnar, A<br>2011 [72]	Drying effects on fig nutrients and PCs	Comparison of drying methods (oven vs sun drying)<br>Two harvesting times<br>Organic acids and sugars<br>PCs: TPC<br>HPLC-MS<br>AOX: DPPH	1 fig variety	Slovenia	Organic acids and sugars high in dried figs compared to fresh figs<br>The predominant phenolic compound was epicatechin in all samples<br>Oven drying provided higher concentration PCs compared to sun dried samples

Caliskan O,<br>2011 [25]	Phytochemical &<br>AOX properties	PCs: TPC, TAC<br>HPLC<br>AOX: FRAP<br>sugar analysis<br>skin color analysis	76 Fig accessions<br><br>Diff colors:<br>green<br>yellow<br>brown<br>purple<br>black	Turkey	Black figs higher AOX, TPC, TAC compared to other fig accessions<br>Fig accessions displayed variable TPC, AOX, and TAC profiles depending on fruit skin color<br>Fructose& glucose predominant sugars<br>Good correlation between the TPC & AOX
Pande G,<br>2010 [47]	Organic acids, sugars, fatty acid profile and PCs	PCs: TPC<br>HPLC<br>AOX: FRAP, TEAC<br>organic acids, sugars: HPLC<br>fatty acid profile: GC-FID<br>total carotenoids, phytosterols, tocopherols, phospholipids	1 fig variety<br><br>peel, pulp containing minute<br>inseparable seeds, and edible portion (whole fruit)	USA	Major organic acid: malic acid<br>Major phenolic acid: gallic and ellagic acid<br>Major flavonoid: catechin<br>Predominant fatty acid: Linoleic acid

Oliveira AP, 2009 [38]	Phytochemical composition and biological potential	PCs and organic acids: HPLC-DAD/UV<br>AOX: DPPH, O_2^-, NO<br>Antimicrobial<br>acetylcholinesterase inhibitory capacity	2 fig varieties<br>White<br>Leaves, peels & pulp	Portugal	7 PCs characterized<br>Similar phenolic profile in leaves, peels & pulp samples<br>Organic acids in leaves: oxalic, citric, malic, quinic, shikimic and fumaric acids<br>Organic acids peels and pulps: all of the above except quinic acid<br>Only leaves exhibited AOX against O_2^- radical<br>None of the samples exhibited antimicrobial and acetylcholinesterase inhibitory capacity
del Caro A, 2008 [27]	Polyphenol composition	PCs: HPLC-DAD	2 fresh fig varieties<br>Black & green<br>Peel and pulp	Italy	PCs highest in peels and black variety<br>Rutin present in high amounts in peel of both varieties<br>C3R and C3G- anthocyanins in peel of black variety<br>Chlorogenic and cinnamic acids: present in peels of both varieties<br>Benzoic acid & catechins not detected.
Piga A, 2008 [26]	PCs analysis in pulp & peel	PCs: HPLC-DAD	2 fig varieties<br>Black & white<br>Peel and pulp	Italy	PCs highest in black fig variety and concentrated in the peels especially flavonols and anthocyanins<br>Pulp: anthocyanins as the only PCs detected<br>Catechins: not detected in any variety
Veberic R, 2008 [28]	Fruit quality	Sugars, organic acids	3 fresh fig varieties	Slovenia	No differences in summer & fall harvest crops<br>No differences in sugar & organic acid in 3 varieties

	different harvest times	PCs: TPC, HPLC<br>AOX: DPPH	1 white & 2 purple		Dark varieties had higher AOX & TPC
Veberic R, 2008 [29]	Phytochemical composition<br>different harvest times	PCs: HPLC-PDA	3 fresh fig varieties<br>1 white & 2 purple	Slovenia	Dark skinned varieties had higher PCs<br>PCs in fruits of 2nd crop > than first crop<br>Conc. of PCs: rutin>(+)-catechin>chlorogenic acid>(−)-epicatechin>gallic acid>syringic acid
Dueñas M, 2008 [53]	Anthocyanin composition	AC(anthocyanin):<br>HPLC-DAD-MS	5 fig varieties<br>green & dark purple<br>peel and pulp	Spain	15 AC pigments with cyanidin as major aglycone; pelargonidin also detected.<br>Rutinose & glucose major sugars attached to AC. Acylation with malonic acid also observed<br>AC derived pigments detected<br>AC > in skin compared to pulp<br>C3R>C3G both in skin & pulps<br>Malonyl derivatives: skin>pulp
Solomon A, 2006 [30]	AOX	Color analysis<br>PCs: TPC, TAC, TFC<br>AC: HPLC<br>AOX: TEAC<br>NMR	6 fig varieties<br>black, red, yellow & green<br>peel and pulp	Turkey	Darker varieties > TPC than lighter<br>Fruit skins>TPC & AOX than pulps<br>C3R: major AC confirmed by NMR<br>C3R contributed 92% of AOX anthocyanin fraction<br>AOX correlated with TAC and TPC<br>Mission variety had highest TPC, TAC and AOX capacity

Su Q,<br><br>2002 [57]	Carotenoids	Carotenoids analyzed by HPLC-UV	Fig along with other foods<br><br>2-6 specimens	Australia	The carotenoids present in figs included lutein, cryptoxanthin, lycopene, β-carotene<br><br>and α-carotene.<br><br>Fig carotenoids appearing in plasma at very low concentrations
Wendeln MC,<br><br>2000 [80]	Nutritional value of figs	fiber, tannins, lipids, protein, carbohydrates, minerals<br><br>Amino acids (AA): Ion exchange chromatography	14 fig varieties	Panama	pulp had one-third digestible components, mostly carbohydrates lipids & proteins.<br><br>Major AA: leucine, lysine, valine, and arginine, Major minerals: K, Ca, Mg, Na & P<br><br>Small figs had as much nutritional value per gram as large figs.

Arrows: ↓ (decrease), ↑ (increase), ↔ (no effect)

AOX: Antioxidant capacity, ABTS :(2,2'-azino-bis(3-ethylbenzothiazoline-6-sul-fonic acid), AA: amino acids, AC: anthocyanins, AOAC: Association of officials analytic chemist, AAS: atomic absorption spectroscopy, CUPRAC: cupric ion reducing antioxidant capacity , CA: Caffeic acid, CT: Condensed tannin , CC: Carotene content ,DDPH: 2,2-diphenyl-1-picrylhydrazyl, FRAP: Ferric ion reducing antioxidant power, FTIR: Fourier-transform infrared spectroscopy, HPLC-UV : Hight performance liquid chromatography ultraviolet , HPLC-DAD: high performance liquid chromatography diode array detector , HPLC-DAD-ESI/MS: high performance liquid chromatography-diode array detector-electrospray ionization/mass spectrometry, HPLC-RI : high performance liquid chromatography-refractive index, HPLC-PDA : high performance liquid chromatography-photo-diode array detector, LC-DAD-ESI-/MS: liquid chromatography-diode array detector electrospray ionization/ Mass spectrometry , ME : Maceration, MI : maturation index , MEKC : micellar electrokinetic chromatography, NMR: nuclear magnetic resonance , ORAC: oxygen radical absorbance capacity , PCs : phenolic compounds , PAC: Proanthocyanidins , ROS : reactive oxygen species , SLE: solid liquid extraction , SEM: scanning electron microscope , TPC: total phenolic content, TAC : Total anthocyanin content, TFC: total flavonoid contents, TPAC : total proanthocyanidins content, TPA : total proanthocyanidins, TTC : total tannin content, TCC: total carotenoids content, TSS: total soluble solids , TBARS: Thio barbituric acid reactive substances, TEAC : Trolox-equivalent antioxidant capacity, TBHQ: tert-butylhydroquinone, UHPLC-ESI-MS : ultra-high-performance liquid chromatography electrospray ionization mass spectrometry
